# Substation Equipment Defect Detection Based on Improved YOLOv8

**DOI:** 10.3390/s25113410

**Published:** 2025-05-28

**Authors:** Yiwei Sun, Xiangran Sun, Ying Lin, Yi Yang, Zhuangzhuang Li, Lun Du, Chaojun Shi

**Affiliations:** 1 State Grid Shandong Electric Power Research Institute, Jinan 250003, China; lysgwork@163.com (Y.L.); waiwai814@126.com (Y.Y.); 1zzsdu@126.com (Z.L.); dulun8998@163.com (L.D.); 2 State Grid Tancheng Power Supply Company, Linyi 276100, China; sgcctcsxr@163.com; 3 Department of Electronic and Communication Engineering, North China Electric Power University, Baoding 071003, China; scj@ncepu.edu.cn

**Keywords:** object detection, substation equipment, defect detection, YOLOv8

## Abstract

The detection of equipment defects in substations is crucial for maintaining the normal operation of power systems. This paper proposes an object detection algorithm for substation equipment defect detection based on improvements to the YOLOv8 model. First, the backbone of YOLOv8 is replaced with EfficientViT, which not only reduces computational redundancy but also enhances the model’s feature extraction capabilities, thereby improving overall performance. Second, a Squeeze-and-Excitation (SE) attention mechanism module is incorporated at the terminal stage of the backbone network to reinforce channel-wise feature representation in input feature maps. Finally, the Bottleneck component within YOLOv8’s C2f module is substituted with FasterBlock, which significantly accelerates inference speed while maintaining model accuracy. Experimental results on the substation equipment defect dataset demonstrate that the improved algorithm achieves a mean average precision (mAP) of 92.8%, representing a 1.8% enhancement over the baseline model. The substantial improvement in average precision confirms the feasibility and effectiveness of the proposed modifications to the YOLOv8 architecture.

## 1. Introduction

With the rapid development of smart grid construction, substations as pivotal nodes in power systems have their equipment operational status directly influencing grid security and power supply reliability [1,2]. However, influenced by complex environmental conditions (e.g., illumination variations, equipment occlusion, background interference) and prolonged mechanical stress, substation equipment is prone to defects including oil leakage, equipment deformation, structural damage, and abnormal meter readings. These defects pose significant risks to substation safety operations and may potentially trigger power system hazards [3]. Therefore, regular inspection and defect identification for substation equipment hold critical importance in ensuring the secure and stable operation of power systems [4]. Conventional manual inspection methods suffer from limitations such as low efficiency, high operational costs, and subjective biases, whereas computer vision-based automated detection technologies have emerged as a critical solution to address these challenges.

To further enhance the accuracy and efficiency of substation equipment defect detection, while leveraging the inherent advantages of the YOLO series in rapid operation and high precision, this paper proposes an improved YOLOv8 [5] method. The proposed approach replaces the original backbone of YOLOv8 with EfficientViT [6], which effectively reduces computational redundancy and enhances model performance through a novel “sandwich” structural block design and the incorporation of a Cascaded Group Attention mechanism. Additionally, a Squeeze-and-Excitation (SE) attention module [7] is integrated to implement global attention mechanisms, enabling adaptive learning of channel-wise feature importance for improved model representation capability. Furthermore, the conventional Bottleneck in the C2f module is substituted with FasterBlock, which optimizes spatial feature extraction by simultaneously reducing redundant computations and memory access overhead.

In the subsequent sections, Section 2 presents related work by other researchers and the specific YOLOv8 structure. Section 3 highlights three model improvements used in this paper: EfficientViT, the SE attention mechanism module, and FasterBlock. Section 4 details the experiments, including the experimental setup, datasets, evaluation metrics, comparison and ablation experiments, and robustness experiments. Section 5 summarizes and offers prospects for the work and discusses some matters related to practical deployment.

## 2. Related Works

In recent years, deep learning algorithms have advanced rapidly. Object detection methods built on convolutional neural networks (CNNs) are now widely adopted in industrial defect detection. These methods demonstrate strong generalization capabilities, enabling robust performance across diverse scenarios. Additionally, their high detection accuracy ensures reliable identification of defects in complex industrial environments. Depending on the training approach, object detection algorithms can be categorized into two-stage detection algorithms and one-stage detection algorithms. The two-stage algorithms, represented by the R-CNN series, include prominent architectures such as Faster R-CNN [8] and Mask R-CNN [9]. In contrast, one-stage detection algorithms are end-to-end frameworks, primarily represented by the You Only Look Once (YOLO) series.

Currently, deep learning-based object detection algorithms have achieved significant progress in industrial defect detection. Kang et al. [10] enhanced the capability of Faster R-CNN models for substation equipment defect detection by introducing a deep hashing auxiliary branch, though this modification resulted in substantial model parameters and high computational complexity. Yu et al. [11] integrated the Faster R-CNN model with sparse representation classification (SRC) algorithms to achieve fine-grained classification of substation equipment defects, demonstrating improved detection accuracy at the expense of overly intricate procedures leading to slower detection speeds. Yang [12] combined Faster R-CNN with SRC algorithms for substation equipment fault diagnosis, similarly increasing computational burden. Huang et al. [4] proposed an improved YOLOv7-tiny-based detection algorithm that effectively accomplished multi-class defect identification in substations. Luo et al. [13] implemented substation equipment defect detection through data augmentation techniques, enhanced loss functions in YOLOX algorithms, and adaptive spatial feature fusion methods, albeit with increased computational load and prolonged training duration. Dong et al. [14] developed a PHAM-YOLO framework based on YOLOv5, employing a parallel hybrid attention mechanism (PHAM) for meter defect detection, but the incorporation of sophisticated attention mechanisms introduced non-trivial computational overhead. Wang et al. [15] enhanced YOLOv5m with FasterNet for lightweight substation defect detection, achieving 41.0% parameter reduction but only a 0.3% mAP gain versus baselines. Zhang et al. [16] proposed an object detection model called YOLO-SD for detecting various device defects in complex real-world scenarios, but the model still has a high number of parameters. Additionally, Wang et al. [17] employed EfficientFormerV2 as the backbone of YOLOv8 for substation equipment defect detection, achieving a YOLO-ViT integration that significantly enhanced detection accuracy. In references [18,19,20,21,22], similar YOLO-ViT hybrid architectures have been applied to diverse industrial inspection domains, where detection precision was further improved. These results collectively validate the rationality and effectiveness of combining YOLO with ViT in industrial vision tasks.

As a newer iteration in the YOLO [23] series, YOLOv8 maintains the design philosophy of one-stage object detection algorithms while achieving an enhanced balance between computational efficiency and detection accuracy. Compared to its predecessors (YOLOv5 and YOLOv7 [24]), YOLOv8 demonstrates significant improvements in detection performance under complex scenarios through multiple architectural refinements and training optimizations, while supporting multi-task extensions including object detection, instance segmentation, and pose estimation.

**Figure 1:**
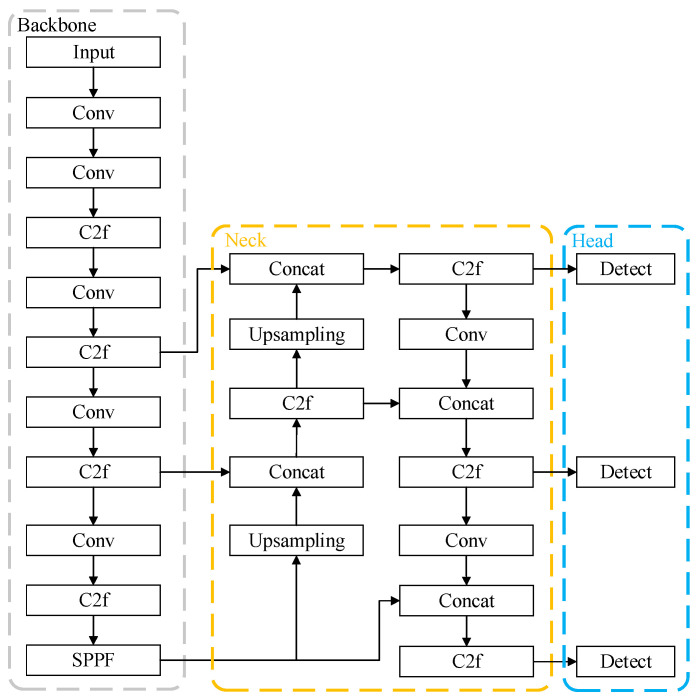
YOLOv8 model architecture.

The architecture of YOLOv8 consists of three components: the Backbone, Neck, and Head, as illustrated in Figure 1. The Backbone is responsible for feature extraction and is based on an improved CSPDarknet framework. It adopts the Cross-Stage Partial Network (CSPNet) [25] structure, with the C2f module serving as the fundamental building unit. Additionally, the Spatial Pyramid Pooling Fast (SPPF) module is integrated to enhance feature representation through multi-scale pooling. Compared to the C3 module in YOLOv5, the C2f module exhibits fewer parameters and superior feature extraction capabilities. The Neck handles multi-scale feature fusion using an enhanced version of the Path Aggregation Network (PANet) [26]. By leveraging a Bidirectional Feature Pyramid Network (BiFPN) [27], it effectively merges shallow-layer details with deep-layer semantic information, thereby improving small object detection and optimizing feature propagation pathways. The Head is tasked with final object detection and classification, comprising a detection head and a classification head. YOLOv8’s detection head employs an Anchor-Free design, directly predicting the target’s center point offsets and width–height dimensions to simplify the model structure and reduce dependency on hyperparameters. Furthermore, it utilizes a Task-Aligned Assigner for dynamic label allocation, which dynamically assigns positive and negative samples based on the alignment between predictions and ground-truth bounding boxes, thereby enhancing training efficiency.

To adapt this framework for the downstream task of substation equipment defect detection, this paper proposes further enhancements to YOLOv8 to optimize model performance.

## 3. Proposed Methods

**Figure 2:**
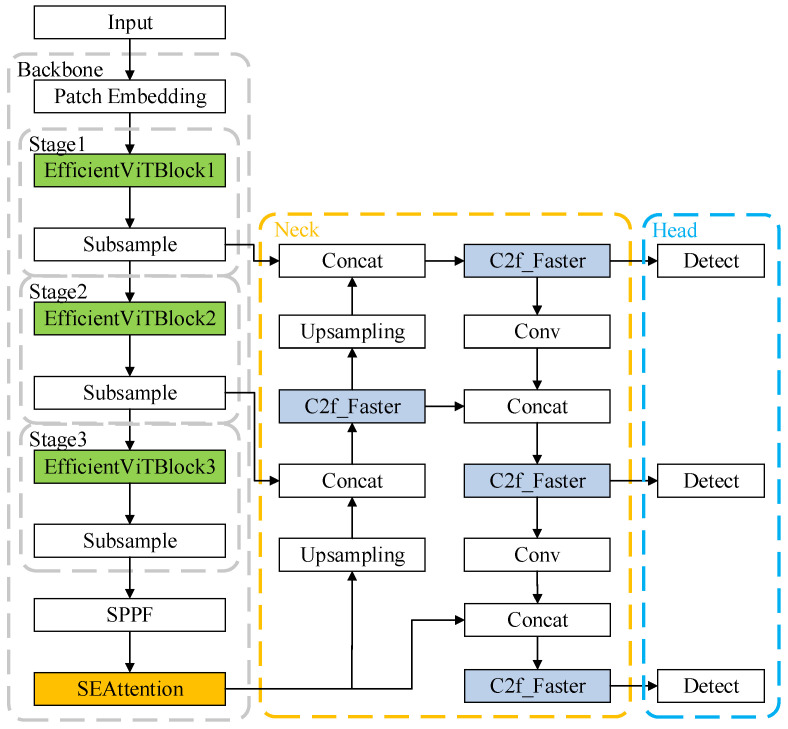
Improved YOLOv8 model architecture.

In order to solve the problem of substation equipment defect detection, this paper proposes a detection network based on YOLOv8 specially designed for this purpose, and its structure is shown in Figure 2. Notably, since YOLOv8 encompasses multiple versions differentiated by layer depth and parameter scale, all improvements in this study are implemented on the YOLOv8n baseline.

As shown in Figure 2, the improved architecture features three key modifications: (1) replacement of the original backbone with an EfficientViT-based backbone to strengthen multi-scale feature representation; (2) integration of an SE attention module at the terminal stage of the backbone to enable channel-wise feature recalibration; and (3) substitution of the C2f module’s Bottleneck components with FasterBlock to optimize computational efficiency. The remaining architectural components retain full compatibility with the original YOLOv8 framework. The method proposed in this paper, while improving the detection accuracy, balances the time cost of training and reasoning to a certain extent.

### 3.1. EfficientViT

EfficientViT is a high-performance vision transformer architecture designed to address the computational inefficiency and high memory footprint of conventional vision transformers [28] while maintaining robust model performance. Traditional transformer models typically employ an equal allocation of multi-head self-attention (MHSA) and feed-forward network (FFN) layers [29] within each block. In contrast, EfficientViT introduces a “Sandwich Layout” that strategically inserts a single MHSA layer between FFN layers. This architectural innovation reduces the temporal overhead of memory-intensive operations in MHSA while enhancing inter-channel communication through expanded FFN layers.

**Figure 3:**
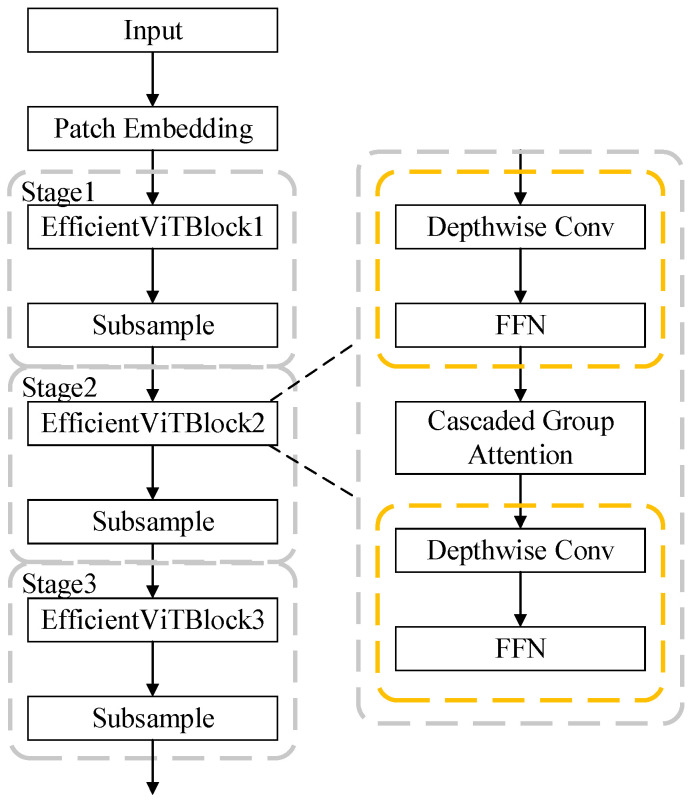
EfficientViT structure.

This paper replaces the backbone of YOLOv8 with an EfficientViT network, whose primary architecture is illustrated in Figure 3. The “Patch Embedding” module serves as the embedding layer, utilizing an overlapping patch embedding layer to partition input images into 16 × 16 patches and project them into feature space. Subsequently, a multi-stage architecture is implemented through three cascaded EfficientViT blocks, interconnected by downsampling layers. Each EfficientViT block comprises multiple stacked EfficientViT units arranged in a sandwich layout, specifically the “Depthwise Separable Convolution (DWConv) [30] + linear FFN + CascadedGroupAttention + DWConv + linear FFN” configuration shown in the right portion of Figure 2. The outputs from these three stages are extracted and fed into the neck network of YOLOv8 for subsequent feature extraction.

### 3.2. SE Attention Mechanism Module

The SE attention mechanism module explicitly models interdependencies among convolutional feature channels to enhance the representational capacity of neural networks. The fundamental principle of this mechanism lies in adaptively assigning channel-wise weights through autonomous network learning, rather than relying on excessive manual intervention as observed in Inception architectures. Functioning as a channel attention module, SE module can enhance the channel features of the input feature maps while maintaining the spatial dimensions of the original input.

**Figure 4:**
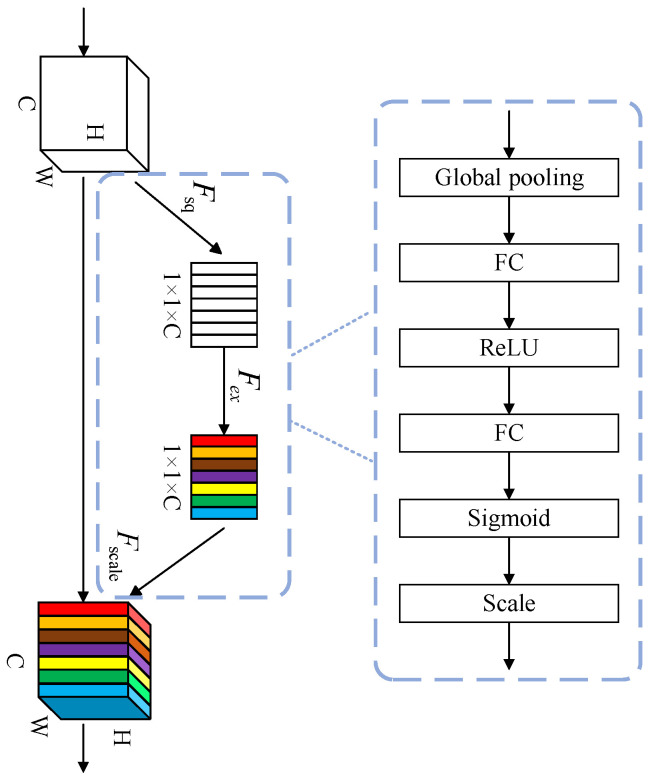
SE module structure.

The SE module operates through three key sequential operations: Squeeze, Excitation, and Scale, with its architectural configuration illustrated in Figure 4. The Squeeze operation aims to compress global spatial information into channel-wise descriptors, effectively capturing the global feature distribution of each channel through global average pooling [31]. Subsequently, the Excitation phase employs a gating mechanism with sigmoid activation [32] to learn nonlinear interactions between channels, thereby generating adaptive channel-wise weights. Specifically, the first fully connected (FC) layer compresses C channels to lower the computational cost. Then, it goes through a ReLU nonlinear activation layer. Next, the second fully connected layer brings the number of channels back to C. Finally, a sigmoid activation function is used to obtain the weights. In the final Scale operation, these calibrated weights are element-wise multiplied with the original feature maps through channel-wise multiplication, producing the refined output of the SE module. This weighted recalibration enables channel-wise attention adjustment by adaptively emphasizing informative features while suppressing less useful ones. In the proposed methodology, SE modules are integrated into the backbone network architecture to enhance channel feature representation, ultimately improving model performance.

(1) Y=SPPF(EfficientNet(X))

(2) SE=SEattention(Y)

where *Y* denotes the output of the input image after passing through *EfficientViT*(·) and *SPPF*(·), and *SE* denotes the output of *Y* after passing through the SE attention module.

### 3.3. FasterBlock Module

The FasterBlock is an efficient neural network module based on Partial Convolution (PConv), primarily designed to optimize feature extraction speed and computational efficiency in vision tasks. Introduced by FasterNet [33], this module aims to significantly enhance inference speed while preserving model accuracy by minimizing redundant computations and memory access.

At its core, PConv operates under the principle of performing spatial feature extraction exclusively on a subset of input feature map channels while preserving the remaining channels unchanged. Specifically, PConv splits input channels into two distinct segments. The first one-quarter of channels (e.g., 16 out of 64) undergo spatial feature extraction using 3 × 3 convolutions. The remaining three-quarters of channels completely bypass any computational processing. This architecture preserves the original data flow for downstream layers to minimize information loss. By selectively processing only a fraction of channels, it reduces memory access operations to one-fourth of traditional convolution requirements. Crucially, this streamlined approach maintains the network’s representational capacity while achieving significant computational efficiency gains. By selectively processing critical channels and leveraging parameter-free identity mappings for others, PConv achieves a favorable balance between computational efficiency and feature discriminability.

**Figure 5:**
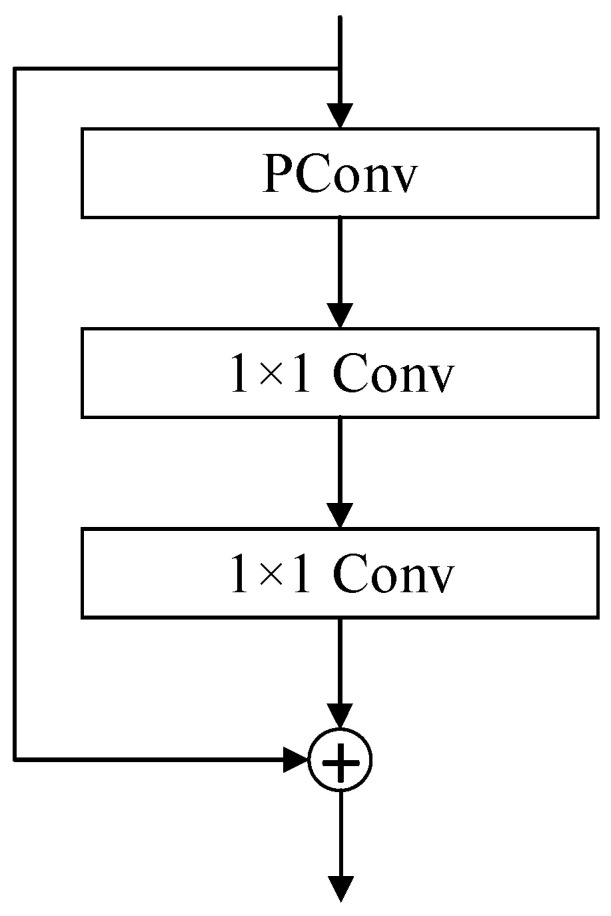
FasterBlock structure.

As illustrated in Figure 5, the FasterBlock comprises a PConv layer and a multi-stage Multi-Layer Perceptron (MLP) [34]. Specifically, the PConv layer performs spatial feature extraction on critical subsets of input channels, while the MLP layer consists of two cascaded 1 × 1 convolutional layers to facilitate inter-channel information fusion and nonlinear transformation. A residual connection is incorporated to preserve original input features through skip connections, thereby mitigating gradient vanishing during backpropagation. The original C2f module employs a computationally heavy Bottleneck structure that depends on stacked 3 × 3 convolutions. In contrast, the FasterBlock introduces an optimized architecture combining PConv and MLP. This redesigned approach achieves substantially lower FLOPs compared to the C2f module. This efficiency stems from PConv’s sparse spatial processing and MLP’s lightweight channel mixing, enabling balanced computation–accuracy trade-offs.

**Figure 6:**
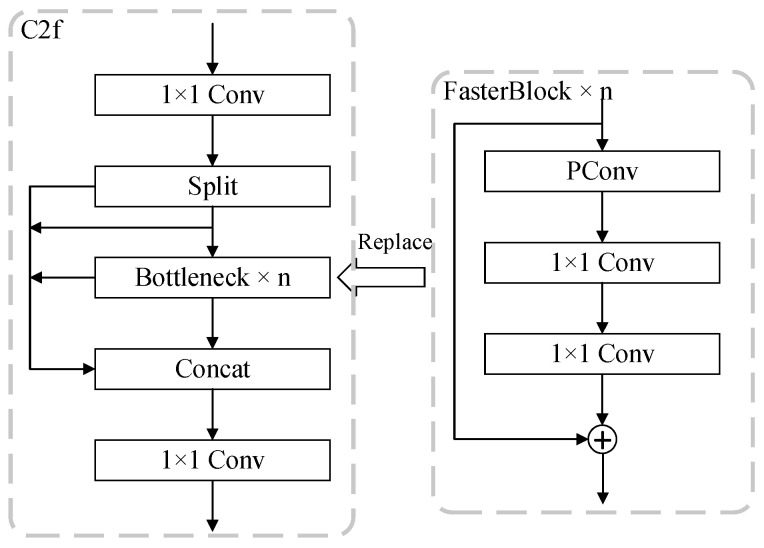
Replace Bottleneck with FasterBlock.

As mentioned above, as depicted in Figure 6, this work replaces the Bottleneck components in the C2f module with FasterBlocks.

## 4. Experiments

### 4.1. Experimental Setup

**Figure 7:**
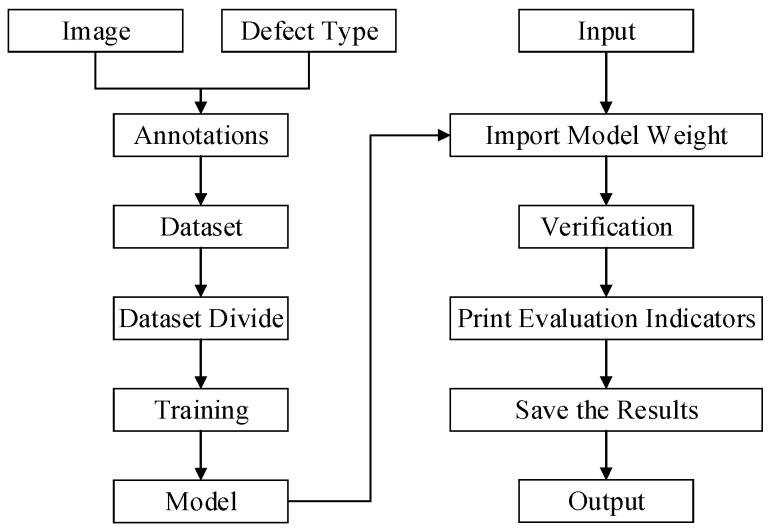
Overall experimental flowchart.

The experiments were conducted utilizing an NVIDIA Tesla P100 GPU under the Linux-based Ubuntu 20.04.4 LTS operating system. Python (version 3.9) served as the programming language implementation, with PyTorch (version 1.11.0) employed as the deep learning framework accelerated by CUDA 11.3. For hyperparameter configuration, the batch size was set to 32 across 500 training epochs, while the learning rate underwent adaptive adjustment through a dynamic scheduling mechanism. Input images were resized to 640 × 640 pixels without additional preprocessing modifications. Model detection accuracy was quantitatively evaluated using two established metrics: mAP50 and the mAP50-95 metric. Meanwhile, Figure 7 shows the experimental process more clearly. The left half is the process of model training, and the right half is the process of model verification.

### 4.2. Dataset

The dataset used in this experiment is shown in Table 1, with a total of 2809 images of defective equipment, including 6 defect categories. Since there are relatively few open-source datasets related to the defects of substation equipment on the network, the dataset used in this experiment is self-labeled. The data used in the construction of the dataset came from the equipment defect images collected by the industrial cameras (types include dome camera, pan–tilt–zoom (PTZ) camera and gun camera) in a substation. The dataset format was YOLO format, and the defects were marked in detail. During the experiment, 80% of the dataset was divided into the training set, and the test set and the verification set accounted for 10% each.

### 4.3. Evaluation Indicators

To objectively evaluate the performance of the improved model in substation equipment defect detection, this study employs mean average precision (mAP) as the primary metric for detection accuracy assessment, complemented by Params and FLOPs to quantify model complexity. These metrics directly reflect the computational and structural overhead of the architecture. In order to verify the results more accurately, two indexes mAP50 and mAP50-95 were used to evaluate the model. The mAP calculation integrates Precision (*P*) and Recall (*R*), where Precision denotes the accuracy of defect localization, and Recall evaluates the model’s capability to identify all potential defects. These metrics are formulated as follows:

(3) $$P=\frac{TP}{TP+FP}$$

(4) $$R=\frac{TP}{TP+FN}$$

In the aforementioned formulae, True Positives (*TP*) denote correctly identified defect instances where positive samples are accurately classified as positive. False Positives (*FP*) represent misclassified negative samples erroneously detected as defects, while False Negatives (*FN*) indicate undetected defects where true positive samples are incorrectly classified as negative. Average precision (*AP*), which quantifies detection accuracy for individual defect categories, is calculated as follows:

(5) $$AP=\int_{0}^{1}P(R)dR$$

Finally, *mAP* represents the average accuracy of all label categories. The larger the value of *mAP*, the better the detection performance of the model. The calculation formula is as follows:

(6) $$mAP=\frac{1}{N}\sum_{i=1}^{N}AP_{i}$$

### 4.4. Experimental Results and Analysis

#### 4.4.1. Defect Type Detection Experiment

To validate the detection precision of the improved model across individual defect categories, a comparative analysis was conducted between the baseline model (YOLOv8n) and the improved model. The evaluation employed AP50 as the primary metric, with detailed comparative results documented in Table 2. As demonstrated in Table 2, the enhanced model exhibits improved AP50 scores across all defect categories, achieving a maximum precision of 95.0% for specific defect types.

**Figure 8:**
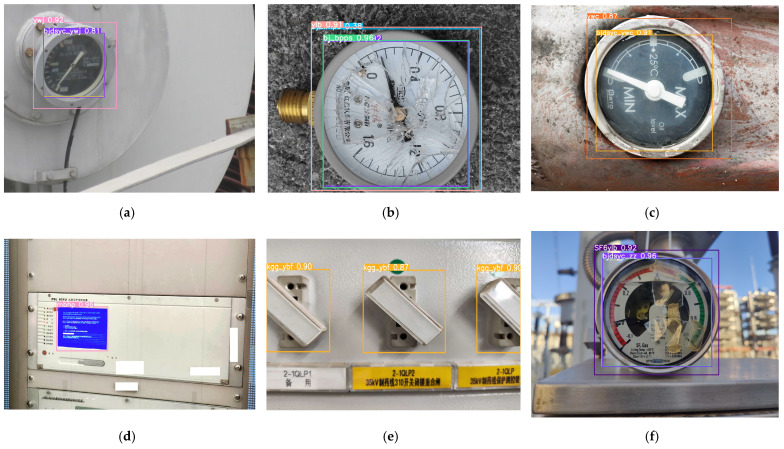
Visual detection results. (**a**) Abnormal oil level gauge reading. (**b**) Damaged dial. (**c**) Abnormal oil level observation window. (**d**) Panel screen. (**e**) Pressure plate is in abnormal state. (**f**) Abnormal pointer reading.

Figure 8 presents visual detection outputs illustrating the model’s capability to effectively localize various defect patterns. Notably, the inclusion of meter-type annotations in the dataset enables simultaneous multi-defect detection and equipment category recognition within individual inspection images.

#### 4.4.2. Ablation Experiment

To validate the effectiveness of individual modules, this study conducts ablation experiments for comparative analysis. Ablation experiments effectively demonstrate the impact of each improvement method on the model’s detection performance, offering critical insights for model design and refinement. The hyperparameter configurations in the ablation experiments remain consistent with the previous experimental setup, with results summarized in Table 3. In the table, “√” indicates that the corresponding improved method was applied to the model, while “--” denotes the exclusion of that particular technique in the modification process.

According to the experimental results presented in Table 3, Improved Model 1 replaces the backbone of YOLOv8n with EfficientViT, achieving a 1.1% improvement in mAP50 compared to the original YOLOv8n. This demonstrates that substituting the backbone network with EfficientViT effectively enhances feature extraction capabilities and model performance without significantly increasing model parameters. Improved Model 2 incorporates an SE attention module at the terminal of YOLOv8n’s backbone network to reinforce channel-wise features of input feature maps. As shown in the table, the integration of the SE module introduces negligible impacts on model parameters and computational costs yet improves average precision by 1.2%. This validates that the SE module strengthens channel-specific feature representations and boosts detection performance without substantially increasing parameter quantity or computational complexity. Modified Model 3 replaces all C2f modules with C2f_Faster blocks in YOLOv8n. Experimental results indicate that adopting FasterBlock reduces both model parameters and computational complexity while simultaneously improving detection accuracy. Furthermore, the proposed improved model integrating all three modifications achieves the most significant performance enhancement, attaining 92.8% detection accuracy. Notably, its parameter count and computational costs are reduced compared to models with only backbone replacement, confirming the effectiveness of our comprehensive optimization strategy. The combined advantages of these three improvements lie in enhancing the model’s detection accuracy while balancing its inference speed, moderately reducing the model’s number of parameters and computational cost. However, the disadvantage is that after introducing the C2f_Faster module, though the number of parameters and computational cost are reduced, the improvement in detection accuracy is not significant.

#### 4.4.3. Comparative Experiment of Different Algorithms

To validate the effectiveness of the proposed improvements, this study conducted comparative experiments with mainstream one-stage and two-stage object detection algorithms. YOLOv3n [35], YOLOv5n, YOLOv6n [36], YOLOv8n, YOLOv9t [37], and YOLOv11n [38] in the YOLO series are selected as the one-stage object detection algorithms, while Faster R-CNN served as the benchmark two-stage detector. The experimental results are summarized in Table 4. As evidenced by the tabulated data, Faster R-CNN, as a two-stage algorithm, exhibits substantially higher parameter counts and computational complexity compared to one-stage counterparts, while underperforming in detection accuracy relative to YOLO series models. Within the YOLO series, YOLOv8n achieves the highest average precision, justifying its selection as the baseline architecture. Notably, our improved YOLOv8n further improves average precision over the original version, demonstrating the effectiveness of the proposed architectural optimizations. In addition, it can be seen from the table that the Frame Per Second (FPS) of the improved YOLOv8n is 238. Although it is lower than that of some other models in the YOLO series, it still meets the real-time requirements.

#### 4.4.4. Robustness Experiment

**Figure 9:**
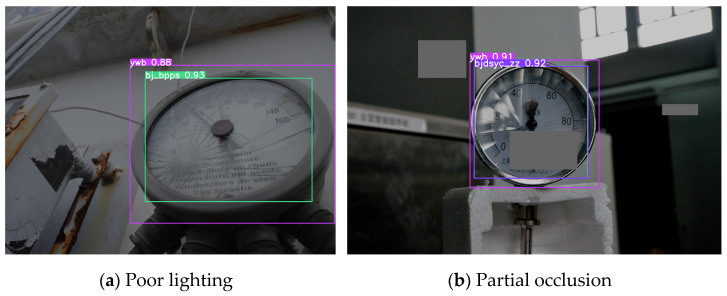
Visualization of robustness assessment and detection.

To evaluate the model’s robustness under different conditions, this study focuses on improving detection accuracy in scenarios of poor lighting and partial occlusion. In one experimental group, the brightness of validation set images was reduced to 50% of the original level to simulate low-light conditions. Another experimental group involved randomly generating three gray rectangular patches (each occupying up to 20% of the image area) at random positions to simulate partial occlusion. The detection performance on processed images is shown in Figure 9, with the left panel demonstrating low-light conditions and the right panel showing partial occlusion.

The robustness evaluation results using modified validation sets are presented in Table 5, which compares detection outcomes between original images, poor lighting conditions, and partial occlusion. The table reveals that poor lighting has a minimal impact on the improved model, while partial occlusion significantly affects detection performance, with most defect categories showing reduced accuracy in robustness experiments compared to standard images. However, the accuracy of some defect categories has still been improved. This might be due to poor lighting or partial occlusion, which reduces the interfering factors in the detection of some images and enables the model to focus more on the recognition of defects.

Table 6 displays the robustness comparison results between YOLOv8 and the improved YOLOv8. The arrows in the table indicate the decrease value of the robustness experiments compared to the mAP trained with normal images. The data demonstrate that under both poor lighting and partial occlusion conditions, the improved YOLOv8 exhibits smaller detection accuracy degradation compared to the original version, indicating enhanced robustness and superior resistance to interference in the modified model.

## 5. Discussion and Conclusions

This paper presents an improved algorithm for substation equipment defect detection based on YOLOv8. The proposed algorithm not only enhances the detection performance by deepening the network architecture and incorporating attention modules but also optimizes the model efficiency by replacing the Bottleneck components in C2f with FasterBlock. This modification effectively reduces the model’s parameter count and computational costs while maintaining detection accuracy, thereby accelerating the inference speed. Experimental results demonstrate that the improved model achieves significantly higher detection accuracy for each defect category in the dataset compared to the original YOLOv8. Furthermore, comparative analysis with other models confirms the superior performance of the proposed model.

However, the proposed method still has certain limitations. For instance, the construction of the experimental dataset may inadequately cover complex scenarios encountered in real-world substation environments, such as extreme illumination variations, dense equipment occlusions, or extreme weather interference. While experimental results demonstrate effectiveness on specific substation equipment datasets, systematic validation of the model’s generalization capability to unseen equipment classes remains unaddressed. Furthermore, current approaches primarily focus on detecting static visual defects, lacking robust spatiotemporal joint modeling capabilities for dynamic defects requiring temporal analysis, such as motion anomalies in mechanical components.

The aforementioned limitations indicate that while current methods have achieved progress in accuracy and efficiency, future research should focus on further optimizing the model’s lightweight design to meet real-time processing requirements. Concurrently, in-depth studies are still needed to enhance environmental adaptability, improve open-set recognition, and explore defect detection methods for broader operational scenarios. These efforts aim to endow the model with stronger generalization capability. In the future, this model could also be extended to detect defects in a wider range of industrial equipment.

Additionally, in practical deployment, the hardware requirements of this model must balance computational capacity with energy efficiency. While the model can be adapted to mainstream edge devices, it necessitates mixed-precision inference support to further optimize real-time performance. Integration with existing systems requires resolving protocol compatibility issues. Key challenges include ensuring model generalization in complex environments (e.g., extreme illumination or occlusion scenarios), which demands enhanced robustness through continuous incremental training and adaptive techniques. Furthermore, constrained computational resources on edge devices may lead to inference latency fluctuations, necessitating optimized resource allocation strategies. Concurrently, deploying AI-based monitoring systems in critical infrastructure such as substations requires rigorous attention to data privacy and system security. Sensitive equipment status data should be transmitted via end-to-end encryption, and multi-layered defense mechanisms (e.g., network isolation) must be implemented to prevent malicious attacks or data tampering.

## Figures and Tables

**Table 1:** The number and types of defect categories included in the dataset.

Defect Type	Label Name	Sample Number
Abnormal oil level gauge reading	bjdsyc_ywj	336
Abnormal pointer reading	bjdsyc_zz	1234
Pressure plate is in abnormal state	kgg_ybf	324
Panel screen	mbhp	342
Damaged dial	bj_bpps	357
Abnormal oil level observation window	bjdsyc_ywc	216

**Table 2:** Comparative experimental results of various defect detection.

Defect Type	Label Name	YOLOv8n (AP50)	Ours (AP50)
Abnormal oil level gauge reading	bjdsyc_ywj	88.8%	89.8%
Abnormal pointer reading	bjdsyc_zz	91.3%	92.4%
Pressure plate is in abnormal state	kgg_ybf	89.8%	90.6%
Panel screen	mbhp	93.4%	95.0%
Damaged dial	bj_bpps	92.0%	93.7%
Abnormal oil level observation window	bjdsyc_ywc	90.6%	95.0%

**Table 3:** Ablation experiment results.

Model	EfficientViT	SE	C2f_Faster	mAP50	Params/M	FLOPs/G
YOLOv8n	--	--	--	91.0%	3.1	8.5
Improved model 1	√	--	--	92.1%	4.1	9.8
Improved model 2	--	√	--	92.2%	3.1	8.5
Improved model 3	--	--	√	91.4%	2.4	6.7
Improved model 4	√	√	--	92.6%	4.1	9.8
Improved model 5	√	--	√	92.3%	3.8	9.1
Improved model 6	--	√	√	92.5%	2.8	7.8
Ours	√	√	√	92.8%	3.8	9.1

**Table 4:** Comparison of different detection algorithms’ experimental results.

Model	mAP50	mAP50-95	Params/M	FLOPs/G	FPS
Faster R-CNN	87.5%	67.3%	41.4	208.3	14
YOLOv3n	89.1%	67.6%	8.8	13.1	119
YOLOv5n	90.2%	70.7%	1.9	4.4	526
YOLOv6n	89.7%	69.8%	4.5	12.8	217
YOLOv8n	91.0%	78.6%	3.1	8.5	322
YOLOv9t	90.5%	73.8%	2.1	7.8	434
YOLOv11n	90.8%	75.7%	2.6	6.5	487
Ours	92.8%	80.7%	3.8	9.1	238

**Table 5:** Experimental results on the robustness of the improved model.

Defect Type	Label Name	Original Images (mAP50)	Poor Lighting (mAP50)	Partial Occlusion (mAP50)
Abnormal oil level gauge reading	bjdsyc_ywj	89.8%	88.5%	87.6%
Abnormal pointer reading	bjdsyc_zz	92.4%	92.1%	90.2%
Pressure plate is in abnormal state	kgg_ybf	90.6%	91.5%	91.0%
Panel screen	mbhp	95.0%	94.7%	96.3%
Damaged dial	bj_bpps	93.7%	91.8%	90.6%
Abnormal oil level observation window	bjdsyc_ywc	95.0%	95.1%	94.6%
All classes	--	92.8%	92.3%	91.7%

**Table 6:** A comparative experiment on the robustness of YOLOv8 and improved YOLOv8.

Image Type	YOLOv8n (mAP50)	Ours (mAP50)
Original images	91.0%	92.8%
Poor lighting	90.3% (↓0.7%)	92.3% (↓0.5%)
Partial occlusion	89.6% (↓1.4%)	91.7% (↓1.1%)

## Data Availability

The datasets presented in this article are not readily available because the data in this article belong to State Grid Shandong Electric Power Research Institute and were used in the Science and Technology project of the State Grid Shandong Electric Power Company. Due to the confidentiality of the project and other reasons, it is not possible to directly access the datasets. Requests to access the datasets should be directed to the corresponding author: sddkysyw@163.com.
